# Comparison of the Effects of Glutamine, Curcumin, and Nesfatin-1 on the Gastric Serosal Surface Neomucosa Formation: An Experimental Rodent Model

**DOI:** 10.1155/2016/2081962

**Published:** 2016-07-21

**Authors:** Osman Bilgin Gulcicek, Ali Solmaz, Hakan Yiğitbaş, Candas Ercetin, Erkan Yavuz, Kamil Ozdogan, Sinan Arici, Asli Kahraman Akkalp, Tulin Sarac, Fatih Çelebi, Atilla Celik

**Affiliations:** ^1^Department of General Surgery, Istanbul Bagcilar Training and Research Hospital, 34100 Istanbul, Turkey; ^2^Department of Pathology, Istanbul Bagcilar Training and Research Hospital, 34100 Istanbul, Turkey; ^3^Department of Biochemistry, Istanbul Bagcilar Training and Research Hospital, 34100 Istanbul, Turkey

## Abstract

*Introduction*. Short bowel syndrome can crop up if more than 50% of small intestine is resected or when less than 100 cm of small bowel is left. Glutamine is the main food source of enterocytes. Curcumin has protective effects on intestinal ischemia-reperfusion damage. Nesfatin-1 is a satiety molecule. It has protective effects on gastric mucosa. The primary purpose of this study is to compare effects of glutamine, curcumin, and nesfatin-1 on the gastric serosal surface neomucosa formation on rats.* Materials and Methods*. 24 Wistar-Hannover rats were randomly divided into 4 groups and treated with saline, glutamine, curcumin, and nesfatin-1 after ileogastric anastomosis. After 14 days all rats were euthanized, and blood was collected. En bloc resection of anastomotic part was performed for histopathological examination.* Results*. PDGF, TGF-*β*, and VEGF levels and neomucosa formation were higher in glutamine group (*p* = 0.003, *p* = 0.003, and *p* = 0.025). Glutamine promotes the intestinal neomucosa formation on the gastric serosal surface and augments growth factors essential for neomucosa formation on rats.* Conclusion*. Glutamine may be used in short bowel syndrome for increasing the absorption surface area. But that needs to be determined by adequately powered clinical trials.

## 1. Introduction

Short bowel syndrome (SBS) is the clinical sequela of inefficient absorption of nutrient and fluid from small intestine that can be congenital or acquired and it is characterized with reduced small bowel length [1]. It is the main cause of intestinal failure (IF) seen after intestinal resection [2] and IF is a term generally used with the SBS [3]. The syndrome is characterized by maldigestion, malabsorption, and malnutrition [4]. Incidence and prevalence are estimated as 3 per million and 4 per million, respectively [5].

The most common causes of SBS are small bowel atresia, aganglionosis, gastroschisis, necrotizing enterocolitis, volvulus, and intussusception in children [6]. Strangulated bowel, Crohn's disease, ischemia, and trauma are the most common factors leading to SBS in adults [4]. SBS is the most common cause of pediatric IF and is associated with significant morbidity and mortality [7]. These patients present symptoms including diarrhea, steatorrhea, abdominal pain, malnutrition, and dehydration. The severity of symptoms depends on the length of small bowel remaining [8]. SBS may crop up after the resection of more than 50% of small intestine and certainly emerges after resection of more than 70% or when less than 100 cm of small bowel is left [9].

The duodenum and jejunum are the primary sites of protein, carbohydrate, fat, water soluble vitamin, and mineral absorption. Half of the jejunum can be removed without significant problem [10]. However resection of the ileum, particularly the terminal ileum, is more detrimental than loss of jejunum, because it is the only site for absorption of intrinsic factor bound B-12 and bile salts [11].

The remaining small bowel undergoes an adaptation process of three phases after resection. The acute phase is the stabilization which starts after resection and lasts less than 4 weeks. The second phase is the adaptation, which lasts 1-2 years. The last phase is the maintenance that requires permanent dietetic treatment [12, 13].

In the final phase of SBS, patients become dependent on long-term parenteral nutrition. However the optimization of intestinal digestion and absorption can be achieved by surgical treatment that consists of reconstructive procedures of remnant bowel and intestinal transplantation [14]. The aim of reconstructive procedures is slowing down the intestinal transit time and the development of new intestinal mucosa, neomucosa. All procedures are still experimental [15].

Glutamine is the primary metabolic fuel of rapidly dividing cells such as small intestinal enterocytes. It also stimulates proliferation of these cells [16, 17]. Previous studies have shown that glutamine has antioxidant effects and immune modulation properties [18]. Curcumin is antioxidant and anti-inflammatory agent whose protective effects on intestinal ischemia-reperfusion damage have been shown in the recent studies [19]. Nesfatin-1 is a recently identified satiety-inducing molecule derived from nucleobindin-2 (NUCB2) in hypothalamic nuclei. It also has anti-inflammatory, antiapoptotic, and protective effects on gastric mucosa [20, 21]. Recently published studies have demonstrated its anti-inflammatory effects via the maintenance of the intracellular antioxidants [22].

The use of serosal patching is a technique to grow new intestinal mucosa. Growing neomucosa is used for enlargement of absorptive surface, but it is still experimental [15].

In this experimental study, our aim was to investigate the potential effects of glutamine, curcumin, and nesfatin-1 on the neomucosa formation of the serosal surface of the stomach that was used as a patch to terminal ileal defect on rats.

## 2. Materials and Methods

### 2.1. Experimental Design

Twenty-four male Wistar-Hannover rats (300–500 g), obtained from Bagcilar Training and Research Hospital Animal Center (BADABEM), were housed in cages under controlled room temperature (21 ± 2°C) and humidity (60–70%) with 12 h light-dark schedule and were fed with standard pellet, ad libitum (MBD Animal Feed, Kocaeli, Turkey). All experimental procedures were approved by the Bagcilar Training and Research Hospital Animal Care and Use Committee (2014-01).

### 2.2. Chemicals and Reagents

The curcuminoid mixture, purchased from Sigma (Sigma, C1386, St. Louis, MO, USA), was identified as curcumin. The authentic curcuminoids were dissolved in corn oil at a concentration of 1 mg/mL in brown glass vials and stored at 4°C. Reagents were obtained from Merck (Darmstadt, Germany). Nesfatin-1 (Bioss, Beijing, China) was dissolved in distilled water and injected intraperitoneally. Glutamine was purchased from Nestle (Resource Glutamine Şase 5 gr; Nestle Healthcare Nutrition, Germany).

### 2.3. Study Groups and Treatment

Rats were randomly divided into 4 groups (8 in each). Group 1 (control) was treated with saline after ileogastric anastomosis between mucosal surface of the ileum and serosal surface of the stomach. Group 2 was treated with glutamine (4 mL/kg/day, by gavage) after the same anastomosis. Group 3 was treated with curcumin (2 mL/kg/day, by gavage) after the same anastomosis. Group 4 was treated with nesfatin-1 (2 *μ*g/kg/day, intraperitoneally). After 14 days all rats were euthanized under anesthesia, and blood was collected by cardiac puncture, centrifuged, and stored at −80°C for the measurement of vascular endothelial growth factor (VEGF), platelet derived growth factor (PDGF), fibroblast growth factor (FGF), transforming growth factor beta (TGF-*β*), and epidermal growth factor (EGF).

**Figure 1:**
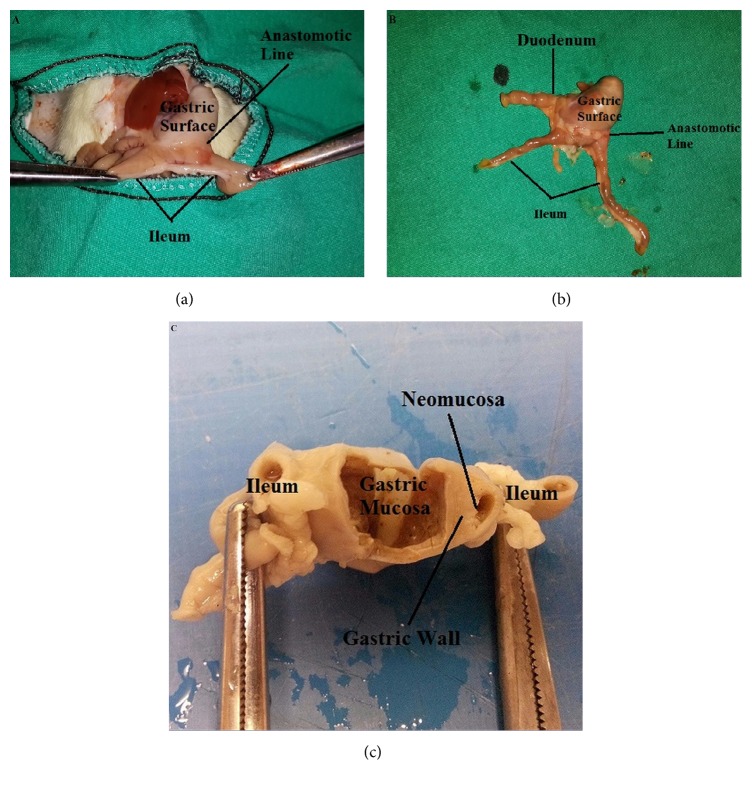
Surgical procedure and histopathological assessment. ((a) and (b)) Anastomotic line is shown between the gastric surface and ileum on the postoperative 14th day. (c) Neomucosa formation on the gastric surface area.

After midline laparotomy en bloc resection of anastomotic part of terminal ileum and stomach was performed and washed with saline and tissues were fixed in 10% formaldehyde solution for histopathological examination (Figure 1).

### 2.4. Surgical Procedure

Rats were anesthetized by an isoflurane (5% for induction and 2% for maintenance, İsoflurane®; Baxter, Puerto Rico, USA). Under aseptic conditions 3 cm midline abdominal incision was done. A one cm longitudinal incision was done in the terminal ileal region and the anastomosis between mucosal surface of the terminal ileum and the serosal surface of the stomach was performed with continuous 6.0 polypropylene sutures (Dogsan, Trabzon, Turkey).

### 2.5. Histological Analysis

**Figure 2:**
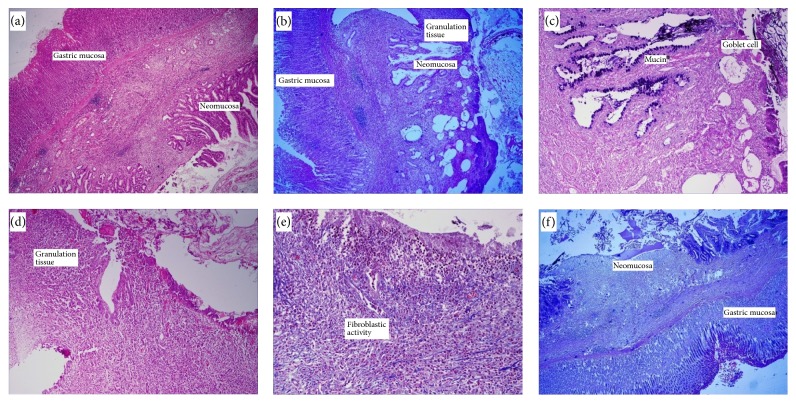
Histological and morphologic evaluation. ((a) and (b)) Gastric mucosa, granulation tissue, and neomucosal tissue formation [hematoxylin and eosin (HE) ×110]. (c) Goblet cells and mucin in neomucosal surface [Alcian Blue (AB) ×220]. (d) Ulcer and granulation tissue in neomucosal surface [HE ×110]. (e) Fibroblastic activity in anastomotic line [Mason Trichrome ×110]. (f) Newly formed neomucosa and gastric mucosa [Alcian Blue ×110].

The anastomotic parts were fixed in 10% formaldehyde and routinely processed for paraffin embedding. Four-micron-thick paraffin sections were obtained and stained with hematoxylin-eosin for the evaluation of inflammatory process, granulation tissue and neomucosa formation, and scoring (0: none, 1: mild, 2: moderate, and 3: severe). Masson's trichrome staining was performed for examination of fibroblastic activity of the neomucosal region, Alcian Blue (pH: 2.5) staining for the examination of intestinal type mucin (Figure 2).

### 2.6. Biochemical Analyses

Blood samples were centrifuged for 10 minutes in 4000 rpm at +4°C. Serum samples were separated into portions and stored at −80°C. Plasma levels of PDGF, VEGF, TGF-*β*, NO, EGF, and FGF were quantified by using enzyme-linked immunosorbent assay (ELISA) kits specific for the rat, according to the manufacturers' instructions and guidelines (Sunredbio, Shanghai, China). These particular assay kits were chosen because of their high degree of sensitivity and selectivity and inter- and intra-assay precision and the small amount of plasma sample required to conduct the assay. Elisa assays were performed with Biotek GEN5 calculation program by using Biotek ELx-800 microplate reader and Biotek ELx-50 microplate washer.

### 2.7. Statistical Analyses

Statistical analysis was performed by using the NCSS (Number Cruncher Statistical System) 2007 Statistical Software (Utah, USA) package program. Descriptive statistical methods (mean, standard deviation), Kruskal-Wallis test (for the group comparison), Dunn's multiple comparison test (for subgroup analysis), and Chi-square test (for comparison of qualitative data) were used. A* p* value of <0.05 was considered statistically significant.

## 3. Results

### 3.1. Biochemical Evaluation

There were no significant differences between nesfatin-1, curcumin, glutamine, and control groups in terms of EGF and FGF levels (*p* = 0.082, *p* = 0.076) (Table 1). Contrarily, there was significant difference between groups in terms of PDGF, TGF-*β*, and VEGF (*p* < 0.05). PDGF level was significantly higher in glutamine-treated group than others (*p* = 0.013, *p* = 0.025, and *p* = 0.004). Glutamine- and curcumin-treated groups had significantly higher TGF-*β* levels compared to control group (*p* = 0.037, *p* = 0.01); however TGF-*β* levels of nesfatin-1-treated group were similar to control group (*p* = 0.337). There was no significant difference between glutamine- and curcumin-treated groups in terms of TGF-*β* levels (*p* = 0.749). VEGF levels of glutamine- and curcumin-treated groups were significantly higher than control group (*p* = 0.016, *p* = 0.025); however nesfatin-1-treated group had nonsignificant results compared to control group (*p* = 0.749). Glutamine-treated group and curcumin-treated group had similar VEGF levels (*p* = 0.998) (Table 2).

### 3.2. Histological Evaluation

**Figure 3:**
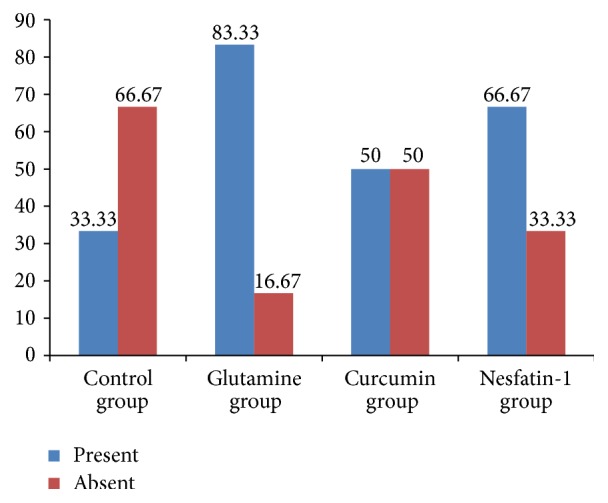
Ratio of neomucosa formation in groups (%).

There was no significant difference between all groups in terms of inflammatory process, granulation tissue formation, fibroblastic activity, and neomucosa formation histologically (*p* > 0.05) (Table 3). Neomucosa formation was determined in 2 rats of control group (33.33%), 5 rats of glutamine-treated group (83.33%), 3 rats of curcumin-treated group (50%), and 4 rats of nesfatin-1-treated group (66.67%) (Figure 3).

## 4. Discussion

Intestinal failure has been defined as the inability of gastrointestinal tract to sustain adequate digestion and absorption without parenteral nutrition. Short bowel syndrome is the most common cause of intestinal failure in children [23]. The management of this syndrome requires a multidisciplinary approach with parenteral nutrition and sometimes surgery [24]. These complex treatment modalities are associated with significant morbidity and mortality rates [25].

SBS is the primary reason for patients to receive long-term parenteral nutrition (PN). PN brings many complications such as life-threatening infections, catheter malfunction, venous thromboembolism, and metabolic complications like liver and renal disease and eventually organ failure [26, 27].

There are several surgical options for the management of SBS, including construction of intestinal valves or reversed intestinal segments, colon interposition, and lengthening procedures [24]. However success rates of these surgical procedures are still limited.

The current surgical approaches for SBS are intestinal transplantation and autologous reconstruction procedures consisting of growing mucosal surface area and lengthening bowel [24]. The main autologous intestinal reconstruction procedures are the Longitudinal Intestinal Lengthening and Tailoring (LILT) known as Bianchi Procedure and the Serial Transverse Enteroplasty (STEP) [28]. Nontransplant surgical approaches (LILT and STEP procedures) are both accepted modalities for the elimination of parenteral nutrition dependence [29]. The LILT procedure was firstly described by Bianchi in 1980. Both procedures have serious complications like stricture, leakage, and bleeding [29].

Intestinal transplantation is the last option for SBS. It has some serious complications and should be performed at specialized centers by experienced surgeons. Morbidity and mortality rates of intestinal transplantation are still very high all over the world [29].

Growing neomucosa is a technique, aiming for augmentation of absorptive surface of the intestinal mucosa; however it is still performed experimentally [15]. The use of serosal patching to grow new intestinal mucosa is a technique used for enlargement of the mucosal surface. The regenerated intestinal mucosa develops by lateral ingrowth of the adjacent mucosa and has same functions as normal intestinal mucosa [30].

There are same experimental models for the growing of the neomucosa by using serosal patch technique in the literature [31]. Serosal surface of the small bowel, colon, and peritoneal surface have been used as a serosal patch [32]. Saday and Mir used intestinal surface as a patch in a rabbit model and the neomucosa grown on the serosal side of the common wall of intestine [33].

According to the review published by Freud and Eshet, urogastrone, octreotide, epidermal growth factor (EGF), and prostaglandin E2 analogues have beneficial effects on the neomucosa formation on the serosal patch [31].

Recently it has been suggested by some authors that transforming growth factor beta (TGF-*β*), growth hormone, and epidermal growth factor (EGF) have favorable effects on bowel mucosa [34]. Glutamine is the primary fuel source for rapidly dividing cells like enterocytes and it prevents intestinal atrophy [35]. Adas et al. showed efficacious effects of hyperbaric oxygen and growth hormone together on the neomucosa formation in the gastric surface patch model [36].

Epidermal growth factor (EGF) is a 53-amino-acid polypeptide which has a mitogenic and cytoprotective role. It stimulates the formation of granulation tissue, epithelial cell migration and proliferation, and formation of angiogenesis [37]. Fibroblast growth factor (FGF) regulates the tissue homeostasis and vascular branching morphogenesis and also increases the levels of transforming growth factor beta (TGF-*β*) [38].

In our study EGF and FGF values of glutamine- and curcumin-treated groups were higher than nesfatin-1 and control groups (Table 1).

Platelet derived growth factor (PDGF) regulates the cellular proliferation and angiogenesis and stimulates the formation of connective tissue matrix, collagen, glycosaminoglycans, and proteoglycans. PDGF promotes the remodeling of soft tissue [39]. Transforming growth factor beta (TGF-*β*) plays an important role in cellular proliferation process, tissue repair, and inflammatory responses. TGF-*β* promotes the expression of type 1 collagen, type 5 collagen, and proteoglycans [40]. Vascular endothelial growth factor (VEGF) stimulates the endothelial cell proliferation and induces angiogenesis. It has an important role in neovascularization [41].

In present study glutamine-treated group had significantly higher levels of PDGF, TGF-*β*, and VEGF. But glutamine and curcumin groups were similar in terms of TGF-*β* and VEGF levels (*p* = 0.74, *p* = 0.99).

Although histopathological examination revealed that there was no significant difference between the groups in terms of inflammatory process, formation of granulation tissue, fibroblastic activity, and neomucosa formation in this study, glutamine-treated group had highest percentage (83.3%, *n* = 5) of neomucosa formation.

Present study is also a surface expander study. And it is supported that intestinal neomucosa can be successfully raised on gastric serosal surface. So regenerative and absorptive capability of intestinal mucosa increase. Also the role of glutamine on intestinal neomucosa formation on gastric serosa was investigated in short bowel syndrome.

In conclusion, glutamine promotes the intestinal neomucosa formation on gastric serosal surface and augments growth factors which were essential for neomucosa formation in rats. Glutamine may be used in short bowel syndrome for increasing the absorption surface area. But that needs to be determined by adequately powered clinical trials.

This study was limited by the small number of rats and short period of the experiment. More meaningful results can be obtained by using a greater number of rats and extending the duration of experiment period. So this study encourages us for further studies.

## Figures and Tables

**Table 1:** Average EGF, FGF, PDGF, TGF-*β*, and VEGF levels (pg/mL).

	Control group	Glutamine group	Curcumin group	Nesfatin-1 group	*p*
EGF	246.39 ± 40.1	293.55 ± 54.09	306.16 ± 45.54	227.08 ± 61.35	0.082
FGF	82.57 ± 8.73	89.07 ± 15.37	94.97 ± 6.11	77.03 ± 14.02	0.076
PDGF	2.38 ± 0.21	3.33 ± 0.52	2.66 ± 0.42	2.1 ± 0.31	**0.003**
TGF-*β*	725.74 ± 89.07	859.21 ± 93.05	877.35 ± 66.08	631.72 ± 166.13	**0.003**
VEGF	264.16 ± 43.5	338.49 ± 50.11	346.6 ± 57.73	261.27 ± 71.75	**0.025**

**Table 2:** PDGF, TGF-*β*, and VEGF levels.

	PDGF (*p* value)	TGF-*β* (*p* value)	VEGF (*p* value)
Control group/nesfatin-1 group	0.055	0.337	0.749
Control group/glutamine group	**0.013**	**0.037**	**0.016**
Control group/curcumin group	0.262	**0.01**	**0.025**
Nesfatin-1 group/glutamine group	**0.004**	**0.01**	0.109
Nesfatin-1 group/curcumin group	**0.037**	**0.004**	**0.025**
Glutamine group/curcumin group	**0.025**	0.749	0.998

**Table 3:** Percentage of inflammatory process, granulation of tissue formation, fibroblastic activity, and neomucosa formation.

		Control group		Glutamine group		Curcumin group		Nesfatin-1 group		*p*
Inflammatory process	Minimal	2	33,33%	2	33,33%	2	33,33%	0	0,00%	0.481
	Mild	2	33,33%	2	33,33%	0	0,00%	1	16,67%	
	Moderate	2	33,33%	2	33,33%	4	66,67%	4	66,67%	
	Severe	0	0,00%	0	0,00%	0	0,00%	1	16,67%	

Granulation tissue form	Minimal	1	16,67%	2	2,00%	2	33,33%	0	0,00%	
	Mild	4	66,67%	3	50,00%	2	33,33%	2	33,33%	0.401
	Moderate	1	16,67%	1	16,67%	2	33,33%	4	66,67%	

Fibroblastic activity	Minimal	1	16,67%	2	33,33%	1	16,67%	0	0,00%	0.242
	Mild	1	16,67%	3	50,00%	5	83,33%	3	50,00%	
	Moderate	3	50,00%	1	16,67%	0	0,00%	3	50,00%	
	Severe	1	16,67%	0	0,00%	0	0,00%	0	0,00%	

Neo.	Absent	4	66,67%	1	16,67%	3	50,00%	2	33,33%	0.33
	Present	2	33,33%	5	83,33%	3	50,00%	4	66,67%	
